# Supplementary material for: Molecular Players of EF-hand Containing Calcium Signaling Event in Plants
Source: Int J Mol Sci. 2019 Mar 23;20(6):1476. doi: 10.3390/ijms20061476 (PMC6471108; doi:10.3390/ijms20061476)
Supplement: Supplementary file 1 [file ijms-20-01476-s001.pdf]

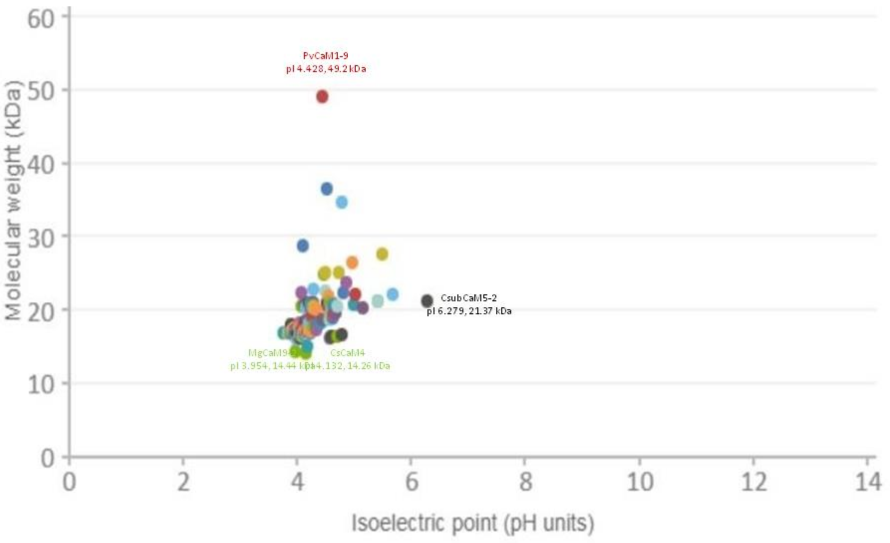

Figure S1. Molecular weight and isoelectric point of CaM Proteins.

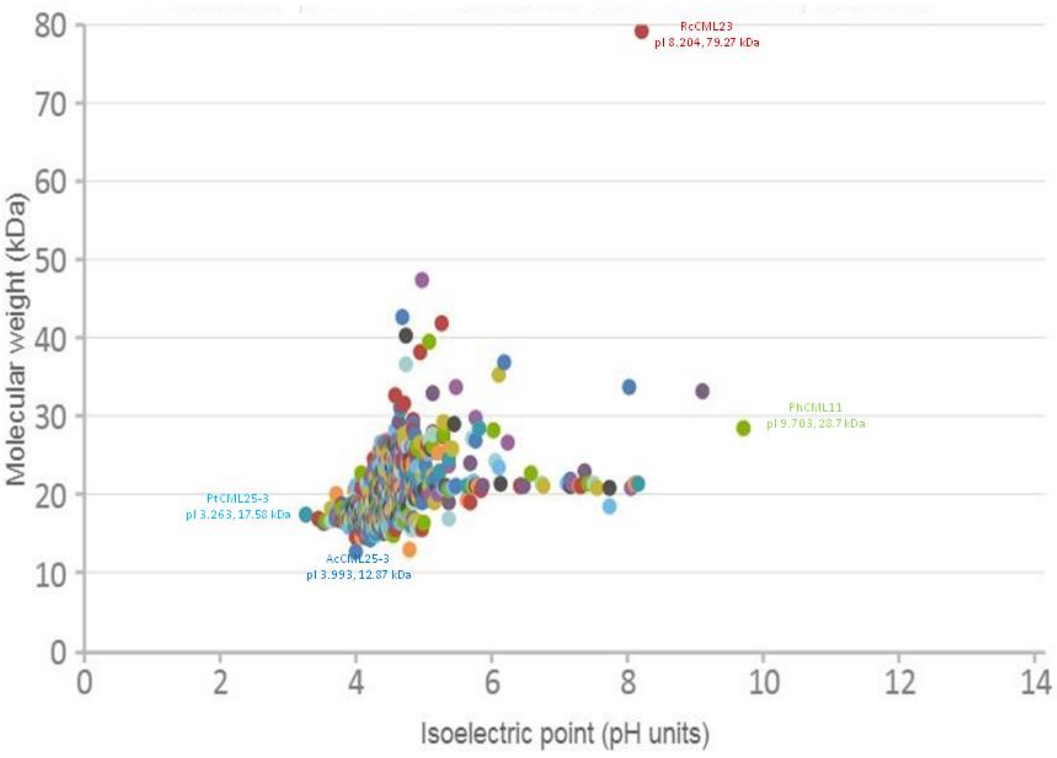

Figure S2. Molecular weight and isoelectric point of CML Proteins

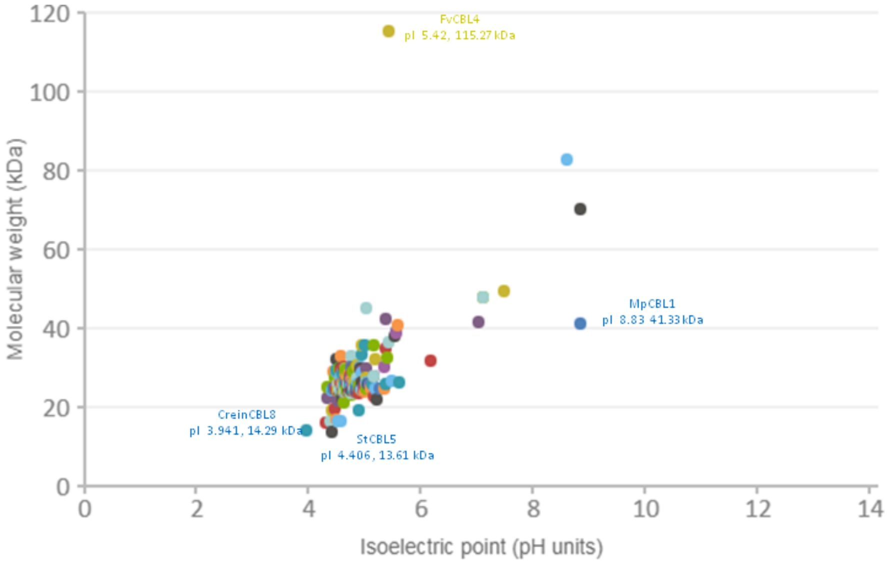

Figure S3. Molecular weight and isoelectric point of CBL Proteins

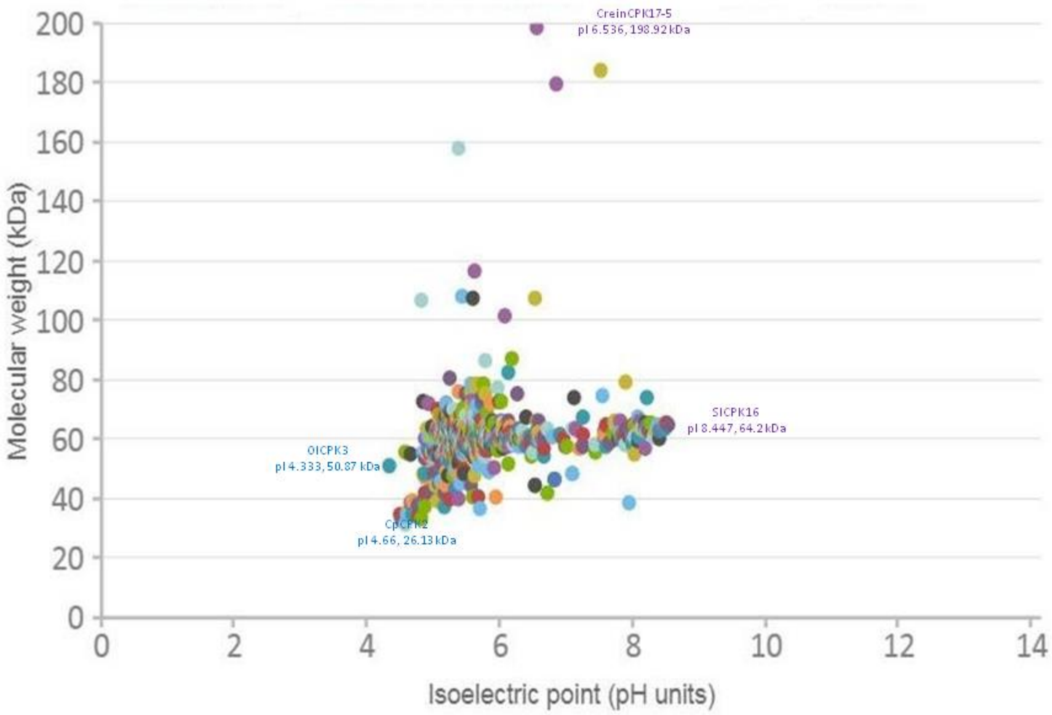

Figure S4. Molecular weight and isoelectric point of CPK Proteins
